# Supplementary material for: In Silico Analyses Indicate a Lower Potency for Dimerization of TLR4/MD-2 as the Reason for the Lower Pathogenicity of Omicron Compared to Wild-Type Virus and Earlier SARS-CoV-2 Variants
Source: Int J Mol Sci. 2024 May 17;25(10):5451. doi: 10.3390/ijms25105451 (PMC11121871; doi:10.3390/ijms25105451)
Supplement: Supplementary file 1 [file ijms-25-05451-s001.zip › ijms-2961456-supplementary.pdf]

Supplementary data

In silico analyses indicate a lower potency for dimerization of TLR4/MD-2 as the reason for the lower pathogenicity of Omicron compared to wild-type virus and earlier SARS-CoV-2 variants

In silico analyses of TLR4 binding and dimerization by trimeric spike protein from SARS-CoV-2 Alpha variant (B. 1.1.7) shows 3x no dimerization and 7x dimerization among the top 10 models.

Table S1: Docking of spike protein trimer from SARS-CoV-2 Alpha variant (PDB 7LWU) to the human TLR4/MD-2 complex (PBD 3FXI) calculated using HDOCK software

Summary of the Top 10 Models

Rank	1	2	3	4	5	6	7	8	9	10
Docking Score	-298.00	-295.89	-294.41	-285.93	-285.84	-281.70	-281.03	-278.81	-278.04	-277.45
Confidence Score	0.9507	0.9487	0.9473	0.9381	0.9380	0.9330	0.9322	0.9293	0.9283	0.9275
Ligand rmsd (Å)	270.79	366.85	235.82	324.02	366.64	224.36	363.95	326.27	332.41	342.50
Interface residues	model 1	model 2	model 3	model 4	model 5	model 6	model 7	model 8	model 9	model 10

Figure. S1

No Dimerization: 3x (models 5,6,10)

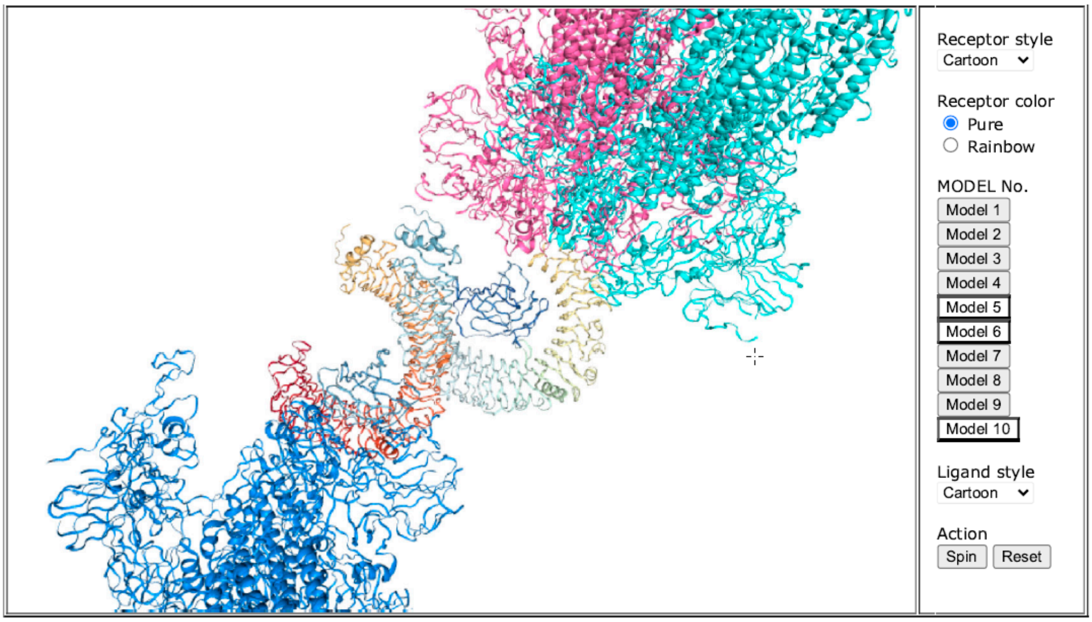

Dimerization: 7x (models 1,2,3,4,7,8,9)

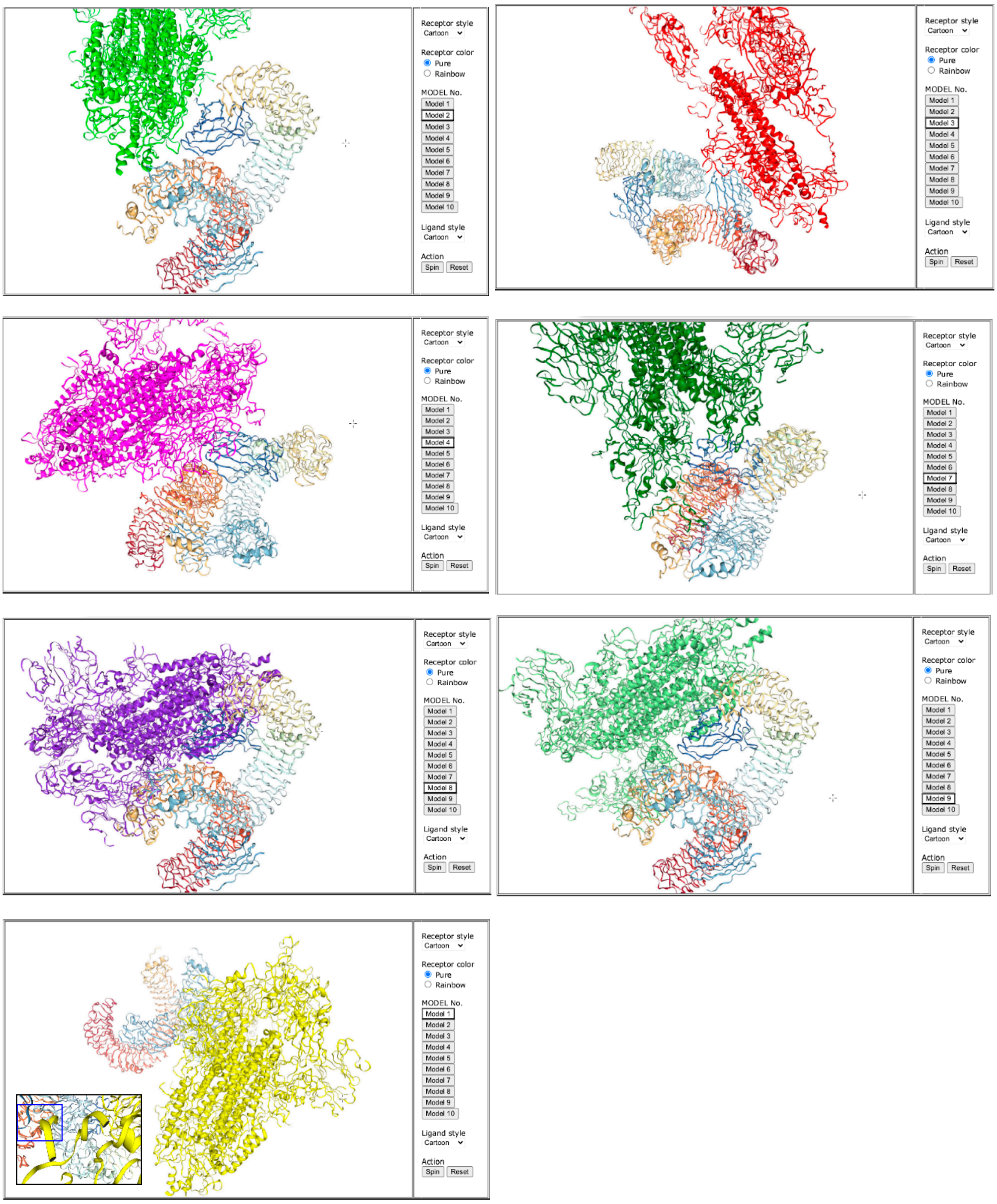

Figure. S1 Top ten docking models for spike protein trimer from SARS-CoV-2 Alpha variant (**PDB 7LWU**) to the human TLR4/MD-2 complex (**PBD 3FXI**) calculated using HDOCK software. Three out of the ten top binding models (models 5,6,10) show binding to TLR4/MD-2 without dimerization, and seven models (models 1*,2,3,4,7,8,9) show binding and dimerization of TLR4/MD-2. *Note:* model 1* shows only a limited dimerization area with MD-2 part (in insert).

In silico analyses of TLR4 binding and dimerization by trimeric spike protein from SARS-CoV-2 gamma variant (P.1.) shows 5x no dimerization and 5x dimerization among the top 10 models.

Table S2: Docking of spike protein trimer from SARS-CoV-2 Gamma variant (PDB 7M8K) to the human TLR4/MD-2 complex (PBD 3FXI) calculated using HDOCK software

Summary of the Top 10 Models

Rank	1	2	3	4	5	6	7	8	9	10
Docking Score	-326.79	-311.39	-305.55	-293.76	-293.17	-292.63	-292.59	-292.21	-290.84	-288.19
Confidence Score	0.9717	0.9619	0.9573	0.9466	0.9460	0.9455	0.9454	0.9450	0.9436	0.9407
Ligand rmsd (Å)	241.93	429.80	362.02	401.06	447.30	273.98	244.34	373.53	346.87	317.53
Interface residues	model_1	model_2	model_3	model_4	model_5	model_6	model_7	model_8	model_9	model_10

Figure. S2

No Dimerization: 5x (models 1,5,7,8,9)

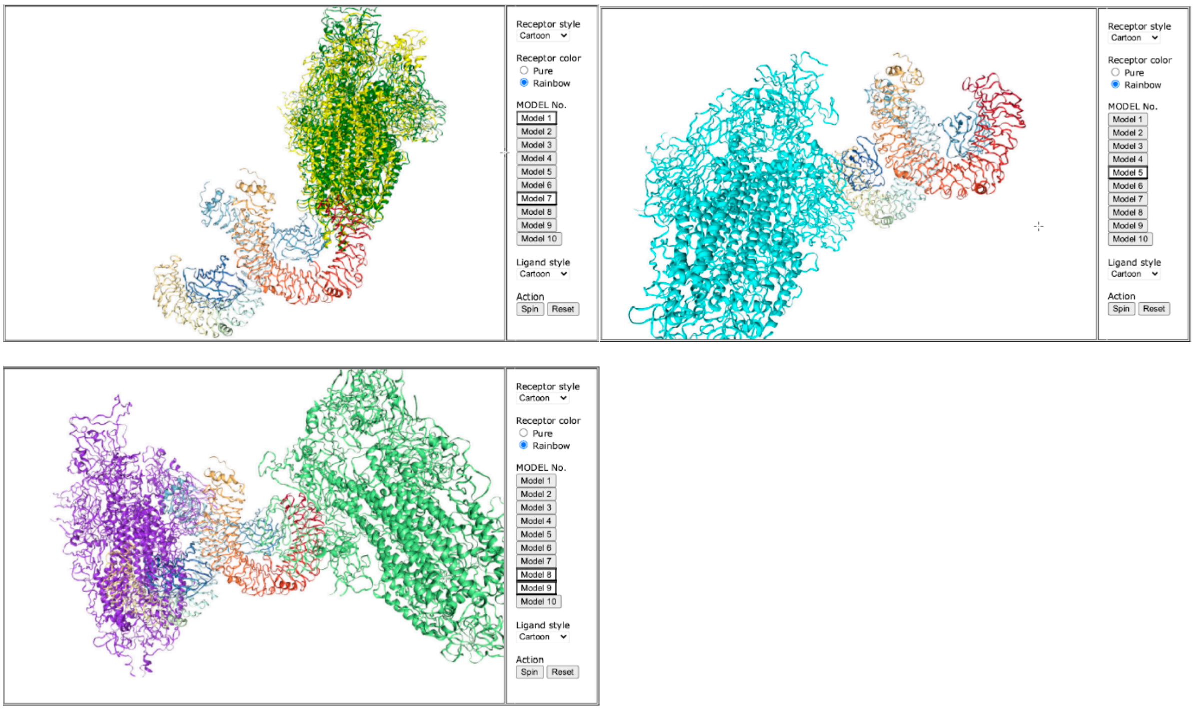

Dimerization: 5x (models 2,3,4,6,10)

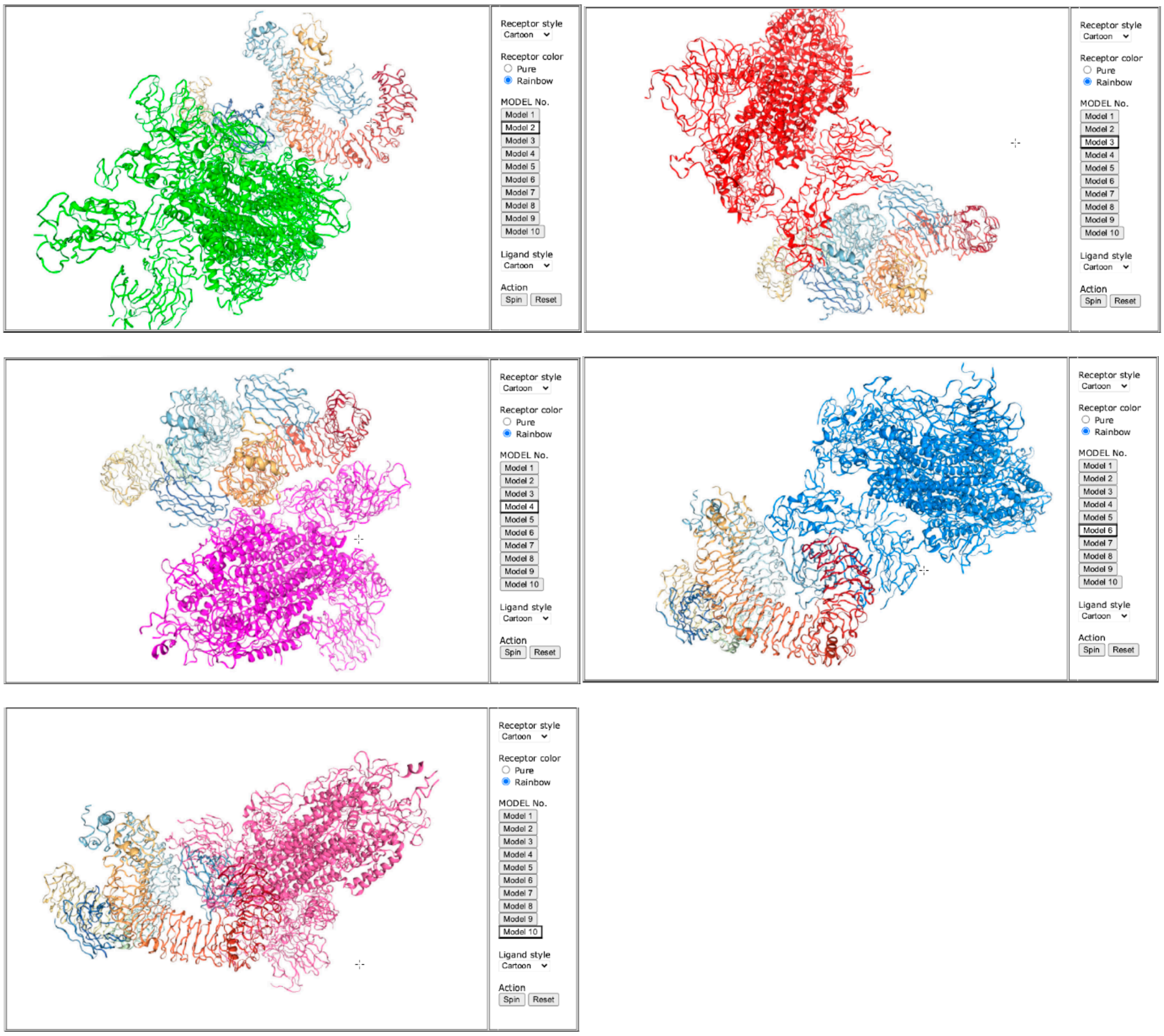

Figure. S2 Top ten docking models for spike protein trimer from SARS-CoV-2 Gamma variant (**PDB 7M8K**) to the human TLR4/MD-2 complex (**PBD 3FXI**) calculated using HDOCK software. Five out of the ten top binding models (models 1,5,7,8,9) show binding to TLR4/MD-2 without dimerization, and five models (models 2,3,4,6,10) show binding and dimerization of TLR4/MD-2.

In silico analyses of TLR4 binding and dimerization by trimeric spike protein from SARS-CoV-2 Delta variant (B. 1.617.2) shows 6x no dimerization and 4x dimerization among the top 10 models.

Table S3: Docking of spike protein trimer from SARS-CoV-2 Delta variant (PDB 2W92) to the human TLR4/MD-2 complex (PBD 3FXI) calculated using HDOCK software

Summary of the Top 10 Models

Rank	1	2	3	4	5	6	7	8	9	10
Docking Score	-332.47	-305.17	-301.82	-299.31	-295.71	-294.62	-294.16	-289.67	-289.36	-289.02
Confidence Score	0.9747	0.9570	0.9542	0.9519	0.9485	0.9475	0.9470	0.9423	0.9420	0.9416
Ligand rmsd (Å)	290.56	342.87	229.99	190.06	254.71	311.02	343.08	259.11	408.49	370.17
Interface residues	model_1	model_2	model_3	model_4	model_5	model_6	model_7	model_8	model_9	model_10

Figure. S3

No Dimerization: 6x (models 1,3,4,5,7,9)

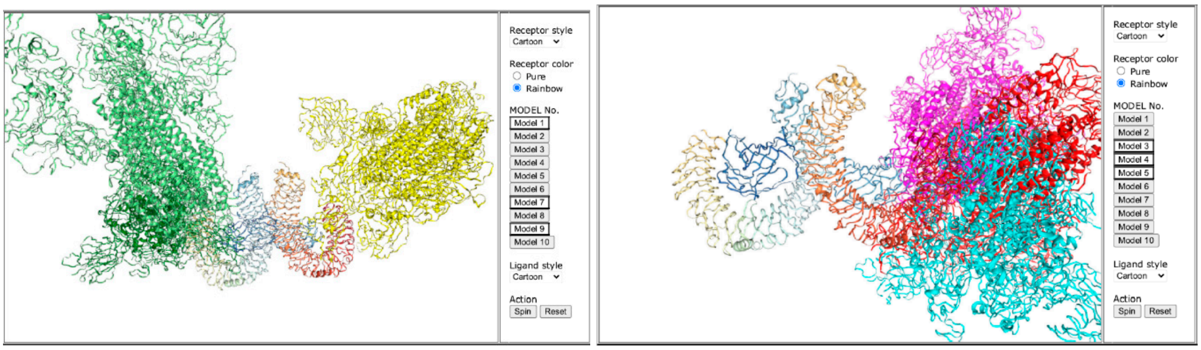

Dimerization: 4x (models 2,6,8,10)

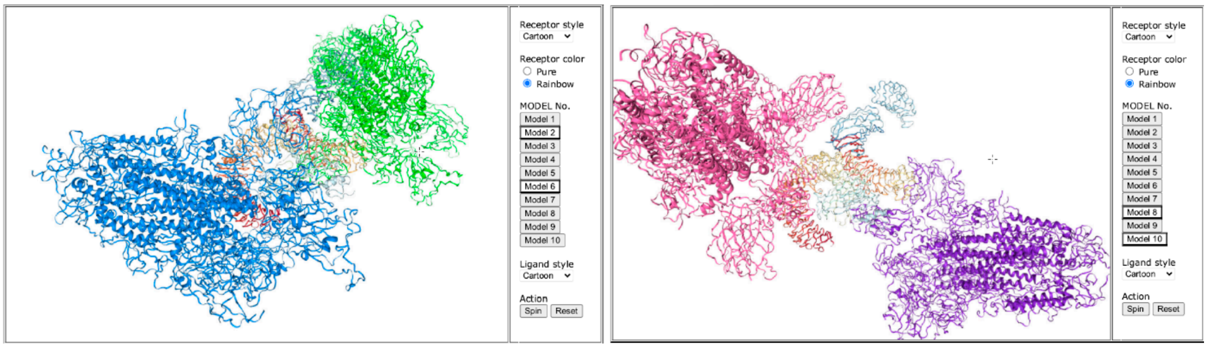

Figure. S3 Top ten docking models for spike protein trimer from SARS-CoV-2 Delta variant (**PDB 2W92**) to the human TLR4/MD-2 complex (**PBD 3FXI**) calculated using HDOCK software. Six out of the ten top binding models (models 1,3,4,5,7,9) show binding to TLR4/MD-2 without dimerization, and four models (models 2,6,8,10) show binding and dimerization of TLR4/MD-2.

In silico analyses of TLR2/TLR1 binding and dimerization by trimeric spike protein from SARS-CoV-2 wild-type shows 3x no dimerization and 7x dimerization among the top 10 models.

Table S4: Docking of spike protein trimer from SARS-CoV-2 wild-type (PDB 6ZGG) to the human TLR2/TLR1 heterodimer (PBD 2Z7X) calculated using HDOCK software

Summary of the Top 10 Models

Rank	1	2	3	4	5	6	7	8	9	10
Docking Score	-257.60	-251.82	-250.84	-248.47	-248.30	-246.59	-244.90	-242.96	-241.80	-241.43
Confidence Score	0.8959	0.8846	0.8825	0.8775	0.8772	0.8734	0.8697	0.8652	0.8625	0.8616
Ligand rmsd (Å)	400.51	311.61	340.05	417.08	328.58	367.99	461.19	406.52	369.65	365.33
Interface residues	model_1	model_2	model_3	model_4	model_5	model_6	model_7	model_8	model_9	model_10

Figure. S4

No Dimerization: 3x (models 2,7,9)

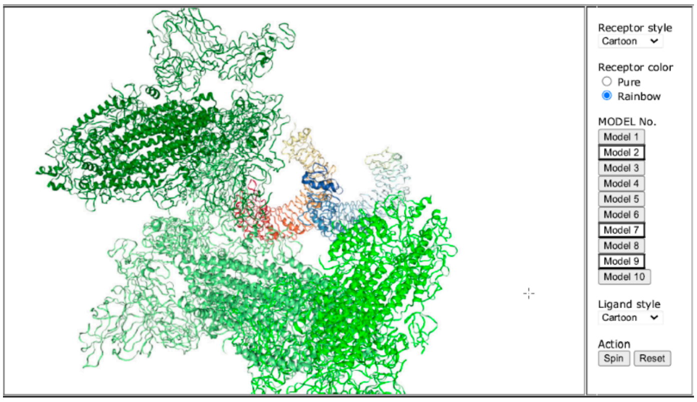

Dimerization: 7x (models 1,3,4,5,6, 8,10)

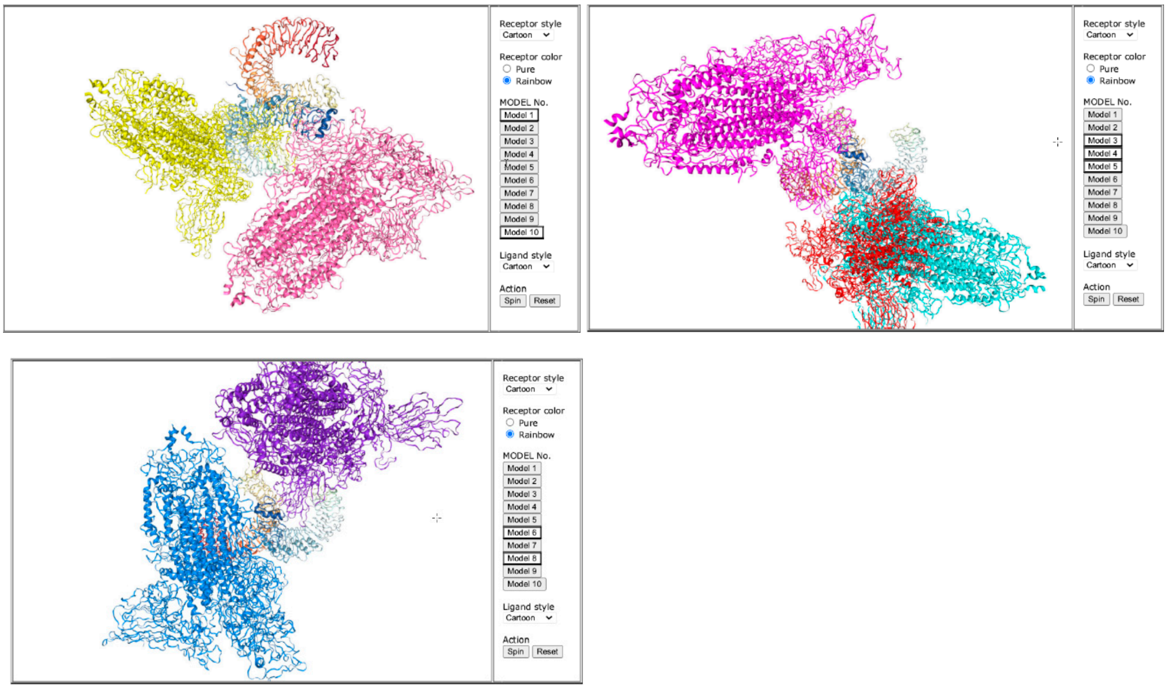

Figure. S4 Top ten docking models for spike protein trimer from SARS-CoV-2 wild-type (**PDB 6ZGG**) to the human TLR2/TLR1 heterodimer complex (**PBD 2Z7X**) calculated using HDOCK software. Three out of the ten top binding models (models 2,7,9) show binding to TLR4/MD-2 without dimerization, and seven models (models 1,3,4,5,6, 8,10) show binding and dimerization of the TLR2/TLR1 heterodimer.

In silico analyses of TLR2/TLR1 binding and dimerization by trimeric spike protein from SARS-CoV-2 Omicron variant shows 2x no dimerization and 8x dimerization among the top 10 models.

Table S5: Docking of spike protein trimer from SARS-CoV-2 Omicron variant (PDB 7TL9) to the human TLR2/TLR1 heterodimer (PBD 2Z7X) calculated using HDOCK software

Summary of the Top 10 Models

Rank	1	2	3	4	5	6	7	8	9	10
Docking Score	-309.81	-305.26	-295.91	-291.52	-284.77	-280.69	-276.53	-268.91	-264.68	-261.99
Confidence Score	0.9607	0.9571	0.9487	0.9443	0.9368	0.9317	0.9263	0.9151	0.9083	0.9038
Ligand rmsd (Å)	378.11	219.28	314.34	302.50	355.66	229.99	233.21	224.19	273.76	323.17
Interface residues	model_1	model_2	model_3	model_4	model_5	model_6	model_7	model_8	model_9	model_10

Figure. S5

No Dimerization: 2x (models 7,8)

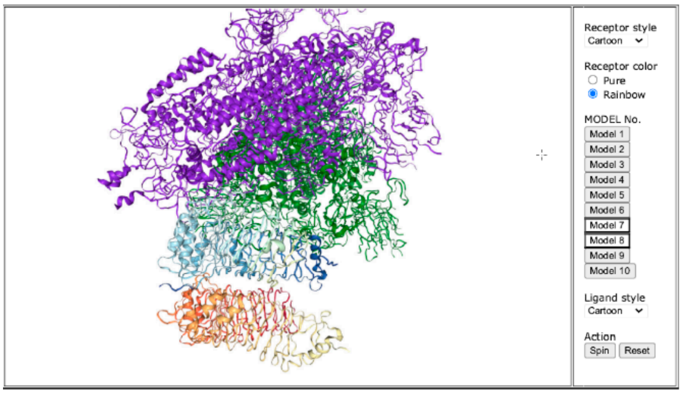

Dimerization: 8x (models 1,2,3,4,5,6,9,10)

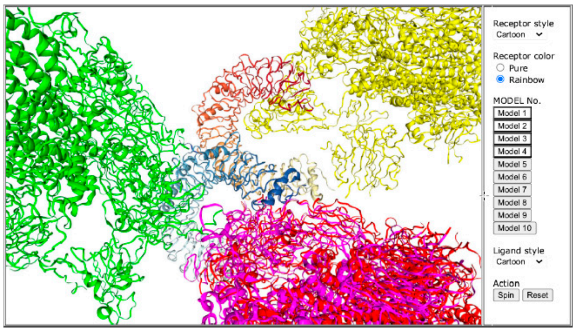

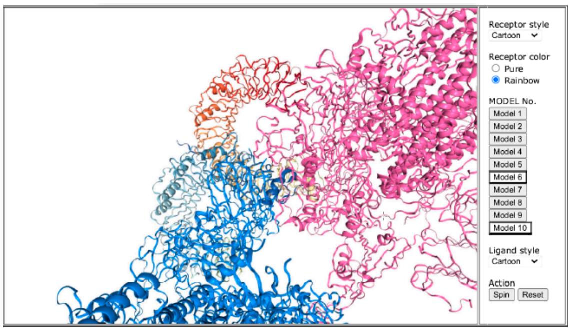

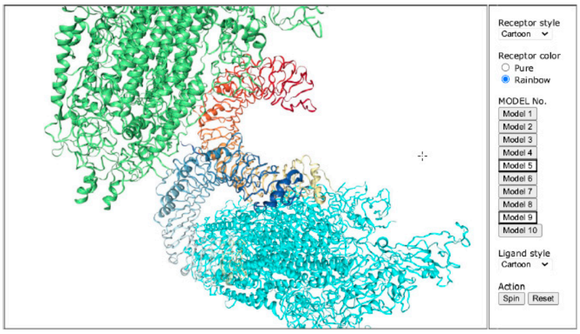

Figure. S5 Top ten docking models for spike protein trimer from SARS-CoV-2 Moicron variant (**PDB 7TL9**) to the human TLR2/TLR1 heterodimer complex (**PBD 2Z7X**) calculated using HDOCK software. Two out of the ten top binding models (models 7,8) show binding to TLR4/MD-2 without dimerization, and seven models (models 1,2,3,4,5,6,9,10) show binding and dimerization of the TLR2/TLR1 heterodimer.

In silico analyses of TLR2/TLR6 binding and dimerization by trimeric spike protein from SARS-CoV-2 wild-type shows 7x no dimerization and 3x dimerization among the top 10 models.

Table S6: Docking of spike protein trimer from SARS-CoV-2 wild-type (PDB 6ZGG) to the human TLR2/TLR6 heterodimer (PBD 3A79) calculated using HDOCK software

Summary of the Top 10 Models

Rank	1	2	3	4	5	6	7	8	9	10
Docking Score	-322.28	-306.00	-298.91	-287.48	-284.98	-283.83	-282.59	-268.06	-267.40	-266.40
Confidence Score	0.9691	0.9577	0.9516	0.9399	0.9370	0.9356	0.9341	0.9138	0.9128	0.9112
Ligand rmsd (Å)	292.14	389.88	295.15	325.30	307.27	337.18	470.90	446.86	354.63	392.55
Interface residues	model_1	model_2	model_3	model_4	model_5	model_6	model_7	model_8	model_9	model_10

Figure. S6

No Dimerization: 7x (models 1,2,3,5,7,8,9)

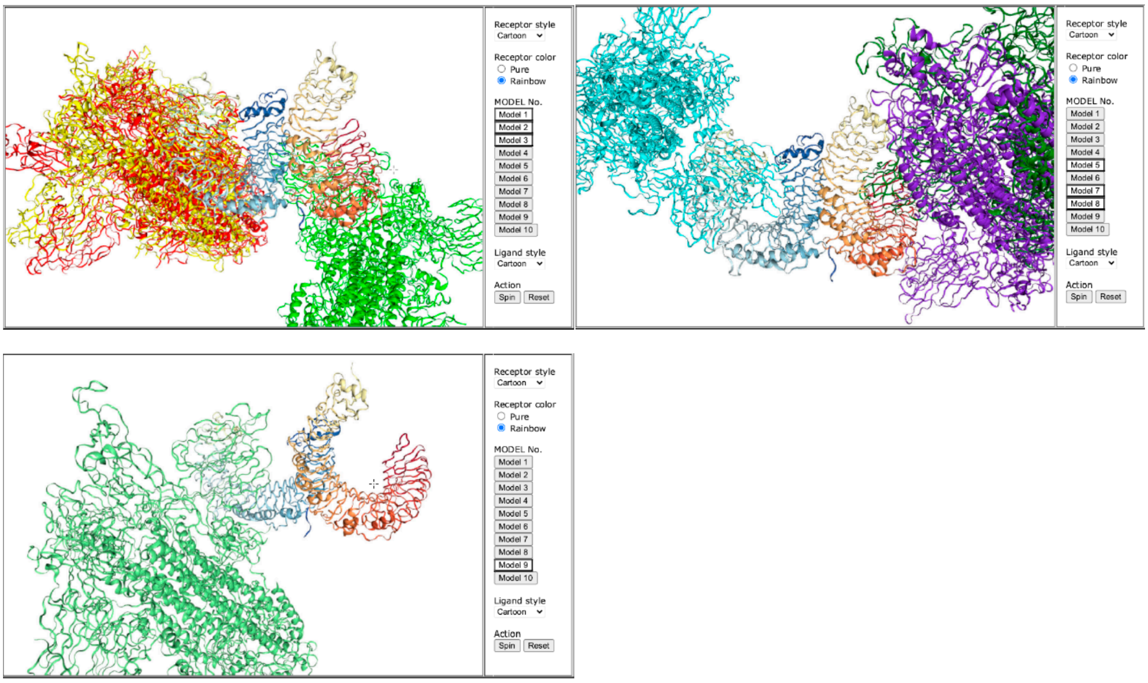

Dimerization: 3x (models 4,6, 10)

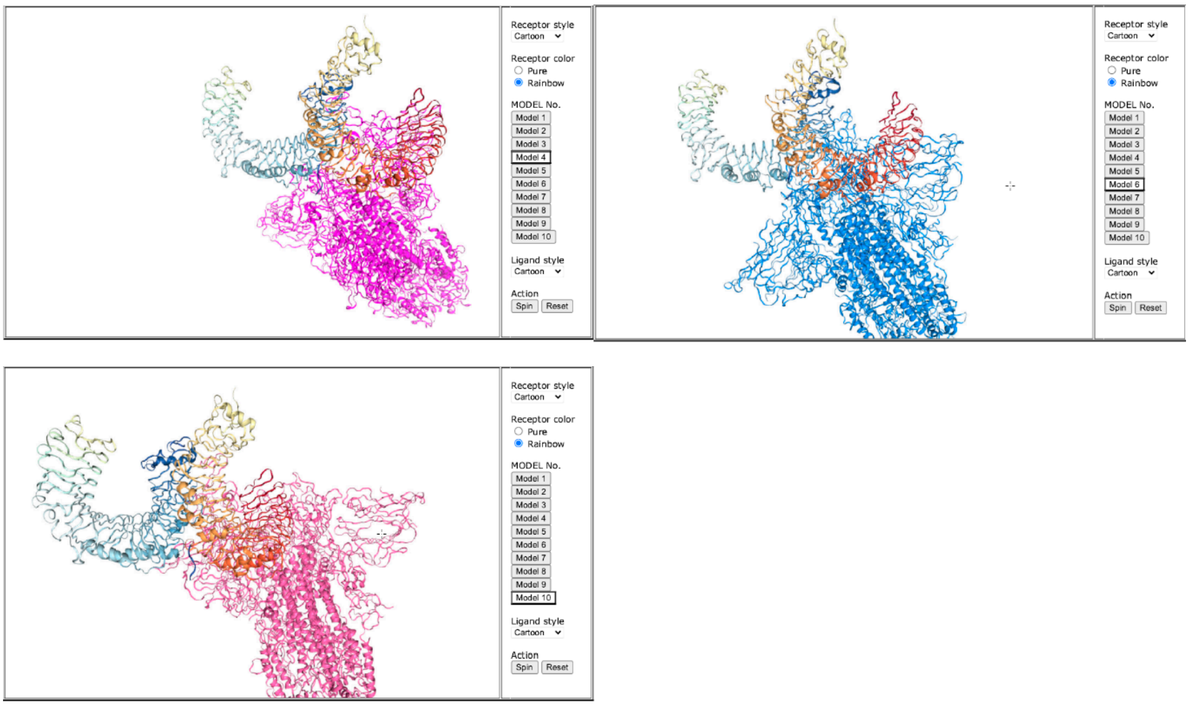

Figure. S6 Top ten docking models for spike protein trimer from SARS-CoV-2 wild-type (**PDB 6ZGG**) to the human TLR2/TLR6 heterodimer complex (**PBD 3A79**) calculated using HDOCK software. Seven out of the ten top binding models (models 1,2,3,5,7,8,9) show binding to TLR2/TLR6 without dimerization, and three models (models 4,6,10) show binding and dimerization of the TLR2/TLR6 heterodimer.

In silico analyses of TLR2/TLR6 binding and dimerization by trimeric spike protein from SARS-CoV-2 Omicron shows 6x no dimerization and 4x dimerization among the top 10 models.

Table S7: Docking of spike protein trimer from SARS-CoV-2 Omicron variant (PDB 7TL9) to the human TLR2/TLR6 heterodimer (PBD 3A79) calculated using HDOCK software

Summary of the Top 10 Models

**Rank**	**1**	**2**	**3**	**4**	**5**	**6**	**7**	**8**	**9**	**10**
**Docking Score**	-329.72	-311.25	-303.38	-301.64	-299.70	-297.63	-297.15	-296.31	-295.06	-290.49
**Confidence Score**	0.9733	0.9618	0.9555	0.9540	0.9523	0.9504	0.9499	0.9491	0.9479	0.9432
**Ligand rmsd (Å)**	276.89	214.24	182.00	246.10	318.34	305.37	351.12	276.19	235.28	351.47
**Interface residues**	model_1	model_2	model_3	model_4	model_5	model_6	model_7	model_8	model_9	model_10

Figure. S7

No Dimerization: 6x (models 1,2,3,4,8,10)

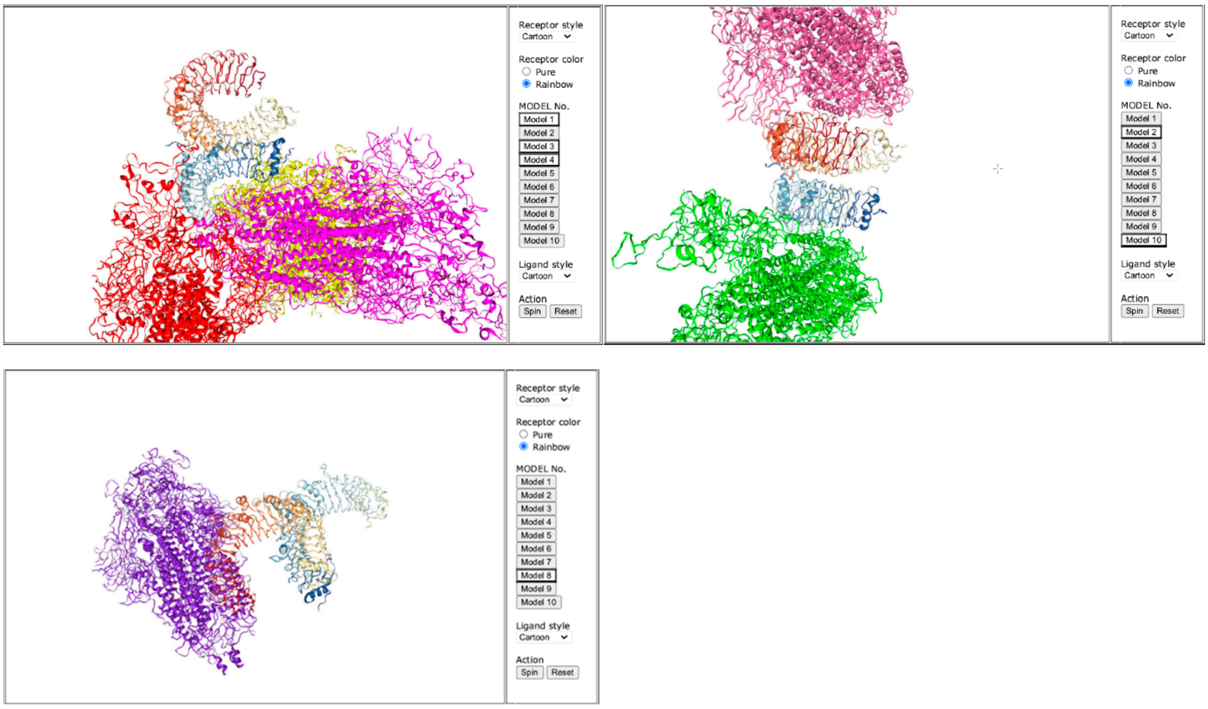

Dimerization: 4x (models 5,6,7,9)

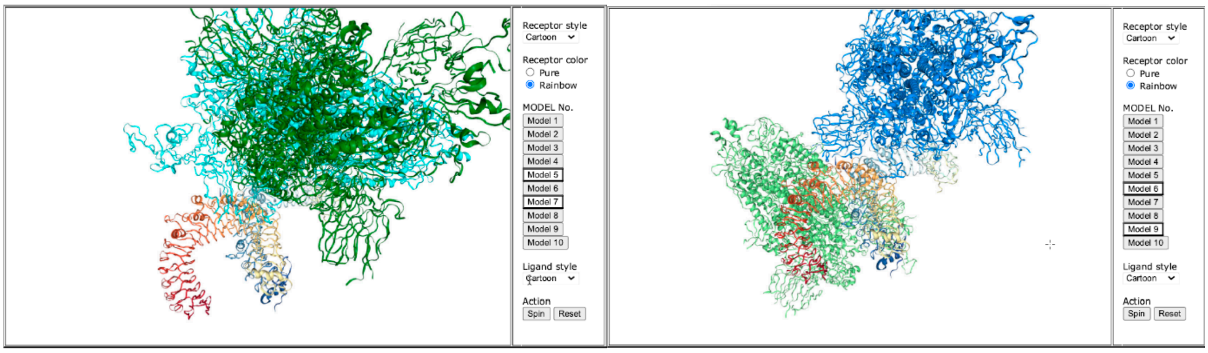

Figure. S7 Top ten docking models for spike protein trimer from SARS-CoV-2 Omicron variant (**PDB 7TL9**) to the human TLR2/TLR6 heterodimer complex (**PBD 3A79**) calculated using HDOCK software. Six out of the ten top binding models (models 1,2,3,4,8,10) show binding to TLR2/TLR6 without

dimerization, and four models (models 5,6,7,9) show binding and dimerization of the TLR2/TLR6 heterodimer.
